# Nonresident Fathers' and Grandparents' Early Years Support and Middle Childhood Socio‐Emotional Adjustment

**DOI:** 10.1111/jomf.12752

**Published:** 2021-01-25

**Authors:** Alison Parkes, Stephanie Chambers, Katie Buston

**Affiliations:** ^1^ MRC/CSO Social and Public Health Sciences Unit, University of Glasgow; ^2^ MRC/CSO Social and Public Health Sciences Unit, University of Glasgow and School of Social and Political Sciences, University of Glasgow

**Keywords:** child development, coparenting, father absence, grandparents, parent involvement, support

## Abstract

**Objective:**

This study investigates how different patterns of nonresident father support for children and mothers in the early years predict middle childhood adjustment, and whether grandparent support has compensating effects.

**Background:**

Nonresident fathers' involvement in children's lives benefits socio‐emotional adjustment, but it is unclear whether support directed at children is compromised by interparental tensions, or whether other factors may compensate for weaker patterns of father support.

**Method:**

Latent class analyses identified patterns of nonresident father support for single mothers and their 34‐month‐old child (None 35%, Low 16%, Moderate 21%, High 28%) and grandparent support (Low 15%, Moderate Maternal 33%, High Maternal 43%, High Maternal and Paternal 9%), using a sample of 648 families from the Growing Up in Scotland cohort. Effects of father support on children's internalizing and externalizing problems from age 46 to 122 months were explored (*n* = 352), together with moderating effects of grandparent support.

**Results:**

Low, Moderate and No father support had similar estimated effects on higher externalizing and internalizing problem levels, and steeper increases in internalizing problems. Compared to Low grandparent support, High Maternal and Paternal grandparent support reduced effects of weaker father support on both types of problem; and was more protective than High Maternal grandparent support against internalizing problems.

**Conclusion:**

Weaker patterns of nonresident father support in early childhood, characterized by low involvement and interparental tensions or by no contact, were associated with poorer middle childhood adjustment. Support from both sets of grandparents offered children most protection against the effects of weaker father support.

Children from single parent households (typically headed by the mother) tend to fare worse across a range of psychosocial outcomes, when compared to those living in two‐parent households (see, e.g., McLanahan et al., 2013; Sands et al., 2017). This difference is attributable to lower parental material and psychological resources, and to conflict surrounding parental divorce or separation (Elam et al., 2016; Pearce et al., 2013; Perales et al., 2016). Nonetheless, ecological and process models (Belsky, 1984; Bronfenbrenner & Morris, 1998) suggest that extra‐familial social support networks can help to alleviate the difficulties faced by single‐parent households. Support from extended family and friends is thought to promote positive psychological states such as self‐worth, and facilitate appraisal and coping in stressful situations (Berkman et al., 2000; Cohen, 2004). In the early years after childbirth, social support increases maternal psychological wellbeing and assists parenting via provision of information and advice, modeling of appropriate behavior, and positive affirmation of parenting skills (Manuel et al., 2012; McConnell et al., 2011). These effects on mothers extend to benefit children's socio‐emotional adjustment (Parkes & Sweeting, 2018).

It is, nevertheless, necessary to recognize the likely limitations to single mothers' benefits from support networks. Support availability may be constrained; both by selection into low‐resource networks (McPherson et al., 2001), and by the need to reciprocate support from nonfamily members (Curry et al., 2013). In consequence, single mothers are often reliant on a small network within the child's extended family (Parkes et al., 2015). A further important limitation stems from the potential of social ties to have negative as well as positive effects on wellbeing, via conflict, insensitivity or perceived interference (Brooks & Dunkel Schetter, 2011). Hence dependence on a small kinship network, especially one containing the nonresident father, may produce strains and conflict that adversely affect single mothers and children.

Our study is concerned with the role of support from nonresident fathers and grandparents for the socio‐emotional adjustment of children from single mother families. It seeks to clarify contextual issues that may modify the impact of nonresident fathering, but which have received little attention (Adamsons & Johnson, 2013). Although much existing work on nonresident father support has considered the father–child dyad in isolation, family systems theory (Minuchin, 1988) signals the need to view the family as a set of interlocking family relationships that jointly shape children's development. Currently, we do not fully understand whether nonresidential father involvement in children's lives has detrimental effects in the context of interparental conflict or uncooperative coparenting (Elam et al., 2016). A systems approach also suggests that a family will recalibrate in times of stress, with its members assuming new roles (Cox & Paley, 1997). Grandparents have been theorized as having a latent function in children's lives, adopting a minor role except in times of crisis (Dunifon & Bajracharya, 2012). It is uncertain, however, whether grandparent support can compensate for any adverse effects of low nonresident father support.

This study uses a person‐centered approach to characterize patterns of early years' nonresident father and grandparent support within a population sample from Scotland, United Kingdom. We investigate how different patterns of nonresident father support are associated with the development of school‐age externalizing and internalizing problems. We also assess whether particular patterns of grandparent support have a protective effect, reducing any adverse effects of weaker nonresident father support on problem development.

## Nonresident Father Support for Child and Mother

Fathers' role in children's socialization is emphasized by theory stressing the value of fathers' *positive* engagement, in addition to indirect care and monitoring of children (Pleck, 2010). A meta‐analysis of nonresident fathers' child‐directed support endorsed this view, finding positive involvement had small positive associations with children's psychosocial development (Adamsons & Johnson, 2013). Yet a potential danger of focusing solely on a father's child‐directed support is that we overlook the part he plays in the extended family system, via the separated couple's continuing relationship and coparenting. Family systems theory and fathering theory view the couple relationship as underpinning family functioning, with coparenting playing a more proximal role in children's development (Bonds & Gondoli, 2007; Feinberg, 2003; Palkovitz & Hull, 2018). Nonetheless, in common with the meta‐analysis of nonresident father child‐directed support referred to above, meta‐analyses of the effects of divorce, interparental conflict or coparenting on child adjustment have found mainly small effect sizes (Teubert & Pinquart, 2010).

In this study, we adopt a holistic definition of nonresident father support encompassing not only a father's child‐directed support (e.g., financial provision and involvement), but also his support for the mother via his contribution to the quality of their relationship and coparenting. This allows us to evaluate whether the combined effects of child‐ and mother‐directed support are more powerful than their known separate effects. It also permits us to address the (as yet, unresolved) question of whether fathers' involvement with children is compromised by interparental tensions. Limited research on older children's adjustment provides apparently contradictory findings: one study found fathers' involvement was more important than interparental conflict (Elam et al., 2016); another using the same sample in later adolescence found the reverse effect (Modecki et al., 2015). Differing findings might reflect the greater durability of particularly acrimonious conflict assessed after a longer postdivorce interval in the second study, but they could also signal possible age differences in effects.

More research is needed on younger children, as nonresident father support is theorized as being most critical in early childhood (Adamsons & Johnson, 2013). Empirical support for this is, however, limited to relatively few cross‐sectional or short‐term follow‐up studies (Adamsons & Johnson, 2013; Choi et al., 2014; Jackson et al., 2015). It is unclear whether benefits are enduring or relatively short‐lived (e.g., in helping families overcome effects of parental separation). Almost all studies involving representative population samples are from the United States (Adamsons & Johnson, 2013): there is a need for more work from countries with alternative welfare systems, where kinship support networks may play a different role. A further critical limitation of existing studies is their exclusive focus on child directed support, using measures of financial provision, frequency of father‐child contact, involvement in the child's activities, and quality of the father–child relationship. Currently, we lack studies that take account of the nonresident father's support for the mother via the interparental relationship and coparenting, as well as his child‐directed support.

## Grandparent Support

Single parent or divorced families are often more reliant than two‐parent families on regular financial, practical, and emotional support from grandparents (Harper & Ruicheva, 2010). In line with this, studies (all of older children and adolescents) suggest grandparent support has stronger benefits for socio‐emotional adjustment among children growing up in lone parent households, compared to those living with both parents (Attar‐Schwartz et al., 2009; Lussier et al., 2002; Ruiz & Silverstein, 2007). There is, however, a lack of research on young children without a resident father. Efforts to clarify the role of grandparents for these children are important, because nonresident father support may be difficult to modify. Some fathers never know their child; for others, support is limited by low economic resources, geographical separation, or re‐partnering (Cheadle et al., 2010; Poole et al., 2015).

Investigation of grandparent support should take account of grandparent lineage. Matrilineal advantage in grandparent relations is a well‐established phenomenon: children, as well as mothers, are usually closer to maternal than to paternal grandparents (Jamieson et al., 2018). Yet there are reasons for supposing that paternal grandparent support may also be important. Paternal grandparent ties are likely to boost the total amount of support available within the family support network, and enhance its flexibility (Doyle et al., 2010). Paternal grandparents may also actively seek to compensate for a father's lack of involvement, and facilitate positive contact between father and child (Doyle et al., 2010; Ryan et al., 2008).

## The Present Study

This study of families draws on data from the Growing Up in Scotland study (https://growingupinscotland.org.uk/), a nationally representative sample of single mother families from Scotland, a country of the United Kingdom. Between 23% and 25% of households with dependent children in Scotland and in the United Kingdom as a whole are headed by a single parent (Office for National Statistics, 2019), usually (90–91% of cases) the mother. In the United Kingdom, social transfers typically form the main element of a single parent's household income, with smaller contributions from employment and child maintenance compared to the United States (Hakovirta & Jokela, 2019). UK family policy modernization reflects an aspiration for nonresident parents to be involved in parenting, in addition to making financial provision for the child (Harvie‐Clark, 2019). In the United Kingdom, grandparents are less likely to be coresident than in the United States (Glaser et al., 2018), but are an important source of early years' childcare (Price et al., 2018).

Our study adopts a “person‐centered” approach, to differentiate groups of families with different support patterns. This allows us to consider support as a “package,” rather than focusing on its separate elements as in traditional variable‐centered methods. From previous person‐centered work, we know that different dimensions of nonresident father and grandparent support do not always align in a consistent manner (see, e.g., Mueller & Elder, 2003; Poole et al., 2015). We use two latent class analyses (LCAs) to identify groups of families with distinct patterns of nonresident father support, and patterns of grandparent support, within our population sample. For nonresident fathers, we consider different patterns of his support for both the child and the mother; and for grandparents we consider both the level and source (paternal and/or maternal) of grandparent support. LCA offers several advantages over standard cluster analysis techniques: these include a model‐based approach to classification on the basis of estimated probabilities, and greater use of formal criteria to decide on the final model (Vermunt & Magidson, 2002).

We evaluate the following hypotheses: (a) low levels of support from nonresident fathers will have positive associations with children's externalizing and internalizing problems; (b) high grandparent support will buffer the effects of low support from nonresident fathers; (c) support from both sets of grandparents (paternal as well as maternal) will have enhanced protective effects, compared to support from maternal grandparents alone.

## Methods

Data were from the first birth cohort of the Growing Up in Scotland study, a nationally representative cohort of families with children born between June 2004 and May 2005 (ScotCen Social Research, 2019). Details of the sampling framework are provided elsewhere (Bradshaw et al., 2007). Data collection was subject to medical ethical review by the Scotland “A” MREC committee. Families throughout Scotland were first interviewed (*n* = 5,217) when children were 10 months old, and followed up at 22, 34, 46, 58, 70, 94, and 122 months (from 2005/2006 to 2014/2015). At each time point, home interviews were conducted with the child's main carer, supplemented by researcher assessments. At 94 and 122 months, information was collected from children using an audio computer‐assisted self‐completion questionnaire, and (at 122 months) from the child's primary school class teacher.

### Samples Used for Analyses of Support Patterns and Child Outcomes

#### Support Sample

To analyze nonresident father and grandparent support patterns in early childhood, we selected the interview time point of 34 months when the most detailed information was gathered. Out of 4,193 families interviewed at this time point, we retained 701 families containing the biological mother, but not the biological father, of a singleton cohort child (exclusions mother not in household *n* = 22, coresident father *n* = 3,459, multiple births *n* = 11). In order to provide a consistent source of information, we then excluded 34 families where the mother had not been interviewed at all of the first three time points, leaving 667 families. We dropped a further 19 families where either the father had died (*n* = 4) or details of contact with the father were not disclosed (*n* = 15), leaving 648 families in the “Support sample.”

#### Analysis Sample

In order to analyze associations between support and child outcomes for families headed by a single mother at 34 months, we excluded families with a coresident partner at 34 months (*n* = 52); and families lacking outcome indicators at 122 months (exclusions, *n* = 244), giving 352 families in the “Analysis sample.”

Sample characteristics are provided in the Table S1. After applying longitudinal survey weights, Analysis and Support samples were closely similar in terms of mothers' educational qualifications, household income, area deprivation and the timing of separation from the father. Both samples contained high proportions of socioeconomically disadvantaged mothers (e.g., 59% of mothers in the Support sample and 58% in the Analysis sample were in the lowest household income quintile). Approximately two‐thirds of mothers either separated from the father before their child was born or were never in a relationship with him (Support sample 69%, Analysis sample 67%).

### Measures

#### Child Outcome Measures: Internalizing and Externalizing Problems

Parent information was collected at child aged 46, 58. 70, 94, and 122 months, and teacher information at 122 months, using the Strengths and Difficulties Questionnaire (SDQ, Goodman, 1997). As recommended (Goodman et al., 2010), externalizing problems combined the 5‐item conduct and attentional problems subscales (Cronbach *α*s for parent reports ranged from .74 to .80, for teacher reports *α* = .85) and internalizing problems combined the 5‐item peer relationship problems and emotional problems subscales (Cronbach *α*s for parent reports ranged from .61 to .79, for teacher reports *α* = .80). SDQ items ask for agreement with statements concerning the child, with responses on a 3‐point scale: 0—not true, 1—somewhat true, 2—certainly true. Example items for externalizing problems are: “Often has temper tantrums or hot tempers,” “Easily distracted, concentration wanders.” Example items for internalizing problems are: “Picked on or bullied by other children,” “Often unhappy, downhearted, or tearful.”

At 122 months only, child self‐reported externalizing problems used one item on misbehaving at school: “How often do you misbehave or cause trouble in class?” Responses were on a 4‐point scale from 1 (never) to 4 (always). Self‐reported internalizing problems used three items on peer victimization (Cronbach *α* = .80) concerning how often other children picked on the respondent “…by calling you names or making fun of you in a way that you don't like,” “…by leaving you out of games and chats?,” “…by shoving, pushing, hitting or picking a fight with you?” Responses were on a 5‐point scale from 1 (most days) to 5 (never) (reverse‐scored for analysis purposes). Child‐reported items were not derived from the SDQ, and were specially designed for the Growing Up in Scotland study.

The main multivariable analyses used externalizing and internalizing scores as continuous measures. Table S6 shows the prevalence of abnormal or borderline abnormal levels of problems after applying recommended SDQ cutoffs (Goodman, 1997).

#### Main Exposure: Nonresident Father Support Class

At 34 months, around a third of fathers (*n* = 223) in the Support sample were not in contact with families. Among the remainder (*n* = 425), three different patterns of nonresident father support were determined using LCA of indicators concerning mothers' reports of the father's involvement with the child (five items concerning his interest in the child and frequency of contact), financial provision (two items: regularity of support, frequency of purchases such as toys or clothing), relationship with the mother (three items: overall relationship, ability to compromise, and disagree calmly), and coparenting (two items: how often parents discussed their child, how often mothers consulted fathers). Details of item wording, response codes and mean scores are provided in a Table S2. To improve interpretability across items with different response scales, all items were recoded as ordered 3‐point categorical variables. For frequency items, cutoffs distinguished support offered weekly (or more often), less frequently, or not at all. For other items, cutoffs distinguished consistent, partial (inconsistent), or generally absent support. The LCA yielded three classes of father support (for details and model fit indices, see Table S3). Combining these classes with the group of families not in contact produced a four‐group typology of father support: None (35%), Low (16%), Moderate (21%), and High (28%). Table 1 shows the distribution of support indicators in the sample of all families in contact with fathers and within each support class. Within each class, fathers' involvement, financial provision, relationship with the mother, and coparenting were strongly positively correlated. Compared to the Support sample, the Analysis sample contained a lower share of fathers not in contact with families (28%), although the distribution of fathers between the remaining three support classes was similar in both samples (Analysis sample: Low 18%, Moderate 25%, High 29%).

**Table 1:** Nonresident Father Support within All Families in Contact When Child Age 34 Months, and According to Support Class

			Nonresident father support class		
		All in contact (*n* = 425)	Low (*n* = 110)	Moderate (*n* = 132)	High (*n* = 183)
Type of support from nonresident father	Level of support	Prop.	Prob.	Prob.	Prob.
**Financial support for child**					
Regularity of provision	Regular	0.58	.41	.47	.77
	Irregular	0.10	.09	.17	.07
	None	0.32	.50	.36	.16
Buys toys, clothes, or equipment	Once a week or more	0.23	.01	.11	.47
	Less often	0.49	.31	.61	.51
	Never	0.28	.68	.29	.02
**Involvement with child**					
Interest in child	Very interested	0.62	.13	.59	.97
	Somewhat interested	0.26	.44	.41	.03
	Not very/at all interested	0.12	.43	.00	.00
Sees child	Once a week or more	0.79	.42	.89	.97
	Less often	0.20	.55	.10	.03
	Never	0.01	.03	.01	.00
Other contact with child (telephone, text message, email or letters)	Once a week or more	0.58	.14	.65	.83
	Less often	0.10	.24	.08	.02
	Never	0.32	.62	.27	.15
Has child to stay overnight	Once a week or more	0.36	.10	.35	.53
	Less often	0.21	.25	.23	.18
	Never	0.43	.66	.43	.29
Takes child on outings or daytrips	Once a week or more	0.47	.12	.39	.76
	Less often	0.32	.34	.45	.21
	Never	0.21	.54	.16	.03
**Relationship with mother**					
Description of relationship	Very/fairly good	0.55	.03	.42	.96
	Neither good nor bad	0.28	.47	.47	.03
	Fairly/very bad	0.17	.50	.11	.01
Parents disagree calmly	Often	0.43	.33	.24	.62
	Sometimes	0.35	.22	.51	.33
	Hardly ever/never	0.22	.45	.25	.05
Parents reach a compromise	Often	0.50	.27	.34	.78
	Sometimes	0.31	.27	.49	.20
	Hardly ever/never	0.19	.47	.17	.02
**Coparenting**					
Parents talk about child	Once a week or more	0.75	.27	.84	1.00
	Less often	0.14	.35	.16	.00
	Never	0.10	.38	.00	.00
Mother asks father for opinion about child	Often/always	0.49	.05	.38	.83
	Sometimes	0.18	.07	.38	.10
	Rarely/never	0.34	.88	.24	.08

Prob. = predicted probability of indicator in Latent nonresident father support class; Prop. = proportion in sample.

#### Potential Moderator: Grandparent Support Class

Mothers supplied information on grandparent childcare (four items: care during the day, babysitting evenings, having the child to stay overnight, taking the child on outings without the mother) and financial support (one item: purchases of toys, clothes or other equipment, other than for special occasions like birthdays). Separate questions were asked about maternal and paternal grandparent support. Details of original items and LCA model fit statistics are provided in Tables S4 and S5. To aid interpretability, the LCA modeled indicators as 3‐point ordered categorical variables coded as weekly, less frequent, or no support. Four patterns of grandparent support identified were: Low (15%), Moderate Maternal (33%), High Maternal (43%), and High Maternal and Paternal (9%). Table 2 shows the distribution of indicators within the entire Support sample and each support class. The distribution of grandparent support classes in the Analysis sample (Low 13%, Moderate Maternal 36%, High Maternal 43%, and High Maternal and Paternal 9%) closely resembled that in the Support sample.

**Table 2:** Maternal and Paternal Grandparent Support within All Families at Child Age 34 Months, and According to Support Class

				Grandparent support class							
		Total sample (*n* = 648)		High Maternal and Paternal grandparent (*n* = 263)		High Maternal grandparent (*n* = 62)		Moderate Maternal grandparent (*n* = 103)		Low grandparent (*n* = 220)	
		Maternal support	Paternal support	Maternal support	Paternal support	Maternal support	Paternal support	Maternal support	Paternal support	Maternal support	Paternal support
Type of grandparent support	Level of support	Prop.	Prop.	Prob.	Prob.	Prob.	Prob.	Prob.	Prob.	Prob.	Prob.
Daytime care for 1+ hours	weekly	0.71	0.06	.77	.58	.98	.00	.69	.00	.01	.00
	less often	0.06	0.02	.10	.17	.01	.00	.12	.00	.01	.00
	never	0.24	0.93	.14	.25	.01	1.00	.19	1.00	.98	1.00
Babysit evenings	weekly	0.47	0.04	.33	.45	.90	.00	.25	.00	.00	.00
	less often	0.19	0.03	.36	.25	.07	.00	.35	.00	.00	.00
	never	0.34	0.93	.31	.30	.02	1.00	.40	1.00	1.00	1.00
Have child to stay overnight	weekly	0.44	0.03	.29	.35	.87	.00	.22	.00	.00	.00
	less often	0.20	0.04	.36	.38	.10	.00	.34	.01	.00	.01
	never	0.36	0.93	.35	.28	.03	1.00	.44	.99	1.00	.99
Take child on outings or daytrips	weekly	0.48	0.04	.36	.47	.87	.00	.29	.00	.00	.00
	less often	0.19	0.03	.35	.28	.10	.00	.35	.00	.01	.00
	never	0.33	0.93	.29	.25	.03	1.00	.37	1.00	.99	1.00
Buy toys, clothes, or equipment	weekly	0.62	0.06	.63	.59	.87	.00	.58	.01	.08	.01
	less often	0.22	0.05	.29	.29	.11	.01	.32	.03	.28	.05
	never	0.15	0.89	.09	.13	.02	.98	.10	.96	.65	.94

Prob. = predicted probability of indicator in Latent grandparent support class; Prop. = proportion in sample.

#### Covariates

Potential confounders of associations between support and child outcomes were (with the exception of partner separation history) measured at the 10 month baseline. All used information supplied by mothers. These included: child's sex, mother's age at the child's birth (<20, 20–29, older), mother's mental health (measured using the SF‐12 scale; Jenkinson & Layte, 1997), family size (1, 2+ children), coresident grandparent, and three indicators of family socioeconomic status. These were (a) mother's education (5‐point scale based on Scottish academic or equivalent qualifications: none, lower level Standard Grades, upper level Standard Grades, Highers and Degree level, where Standard grades were qualifications typically obtained by minimum school leaving age, and Highers were qualifications allowing University entrance); (b) equivalized household income; and (c) deprivation quintile, using the Scottish Index of Multiple Deprivation, a small area measure of relative deprivation across income, employment, education, health, access to services, crime, and housing. Because of the low proportion of ethnic minority mothers (*n* = 7, 1%), ethnicity was not included as a covariate. Partner separation history used information from the interviews at 10, 22, and 34 months to determine when fathers had left the household.

### Missing Data

Missing parent‐reported outcome information averaged 4.7% of Analysis sample cases at any time point, 3.7% of cases lacked child‐reported outcomes at 122 months, and 48% of cases lacked teacher‐reported outcome information at 122 months. There were no differences in parent‐ or child‐reported problems at 122 months, or in the main exposures and covariates, according to the availability of teacher information. We therefore reasoned that although missing teacher information was at high levels, it was not a source of bias. There was complete information on the two main exposures (nonresident father and grandparent support classes), and low rates of covariate missingness (averaging 0.7%, excluding household income where 7% of cases lacked information). Missing outcome information was handled using Full Information Maximum Likelihood in Mplus version 8 45 (Muthén & Muthén, 1998–2017). To include cases with missing covariate information in the models, we declared the variances of incomplete variables (household income, mother's education, mother's mental health). Additional analyses on multiply imputed data sets (*n* = 25) gave closely similar findings, so are not reported here.

### Analysis of Associations between Support and Child Outcomes

We used two types of models, Trajectory and Endpoint, since each offered different advantages. Trajectory models used five repeated parent‐reported measures to explore the development of externalizing and internalizing problems from 46 to 122 months, modeling these as parallel latent growth curves with intercepts at the trajectory midpoint (84 months). Endpoint models focused on outcomes at 122 months, capitalizing on the availability of multiple sources of information at this time point. Here, externalizing and internalizing problems were modeled as latent constructs with parent‐, teacher‐, and child‐reported indicator loadings ranging from 0.5 to 0.8. Since teachers and children provide different insights to those supplied by parents, particularly regarding behavior and feelings outside the home environment, these models offer important additional corroboration and reduce the threat of shared variance.

We investigated possible moderating effects of grandparent support, by adding main effects for grandparent support together with grandparent × nonresident father support interaction terms to both types of model. All analyses used a robust maximum likelihood estimator in Mplus. Endpoint models allowed for the complex survey design and survey weights, although convergence problems permitted allowance only for weights in Trajectory models. Indicator cutoffs applied to assess absolute model fit were ≤0.07 for the root mean square error of approximation (RMSEA) (Steiger, 2007) and ≤0.08 for the standardized root mean residual (SRMR) (Hu & Bentler, 1999). In order to compare the results of Trajectory and Endpoint models, coefficients were standardized with respect to outcomes.

## Results

### Externalizing and Internalizing Problems in the Analysis Sample

The unconditional Trajectory model captured a linear decline in average externalizing problems, and an increase in average internalizing problems, from 46 to 122 months (see Table S6 for average problem scores at each time point). Both problem trajectories had small positive quadratic terms, indicating that the decline in externalizing problems leveled off, while internalizing problems grew faster toward the end of the period.

### Associations between Nonresident Father Support Class at 34 Months and School Age Externalizing and Internalizing Problems

To examine our first hypothesis, we explored associations between nonresident father support at 34 months and problem trajectories, after adjusting for baseline covariates and partner separation history (for unadjusted associations at each time point, see Table S6). When compared to children with High nonresident father support, those in families receiving Moderate, Low, and No support all had higher externalizing and internalizing problems at the 84 month intercepts (coefficient ranges were 0.34–0.72 for externalizing; 0.25–0.47 for internalizing problems, Table 3, Part a). Children in families with Moderate, Low, and No nonresident father support also experienced steeper increases in internalizing problems, compared to those with High support (linear slope terms ranged from 0.35 to 0.56). Overlapping confidence intervals suggest that Moderate, Low, and No nonresident father support had similarly sized effects, although not all were statistically significant (*p* < .05). Endpoint model findings (Table 3, Part b), drawing on outcome information from teachers and children as well as parents, resembled those from the Trajectory model and again suggested similar effect sizes for the three classes of weaker nonresident father support. Thus both types of model supported our first hypothesis.

**Table 3:** Estimated Effects of Nonresident Father Support on Children's Externalizing and Internalizing Problems (*n* = 352)

(a) Trajectory models of parent‐reported problems, child age 46–122 months					
		Externalizing problems		Internalizing problems	
		Intercept		Intercept	
Support (reference)	Contrast	Coefficient (95% CI)	*p*	Coefficient (95% CI)	*p*
N‐res. father (high)	Moderate	0.34 (0.00, 0.69)	.049	0.25 (−0.11, 0.62)	.172
	Low	0.45 (0.07, 0.83)	.022	0.31 (−0.02, 0.64)	.067
	None	0.72 (0.41, 1.02)	<.001	0.47 (0.12, 0.81)	.008
		Linear slope		Linear slope	
		Coefficient (95% CI)	*p*	Coefficient (95% CI)	*p*
N‐res. father (high)	Moderate	0.04 (−0.37, 0.46)	.835	0.56 (0.22, 0.91)	.001
	Low	0.03 (−0.39, 0.45)	.888	0.39 (0.05, 0.72)	.024
	None	0.20 (−0.22, 0.63)	.346	0.35 (−0.03, 0.73)	.067
		Quadratic slope		Quadratic slope	
		Coefficient (95% CI)	*p*	Coefficient (95% CI)	*p*
N‐res. father (high)	Moderate	−0.12 (−1.19, 0.95)	.821	0.22 (−0.92, 1.35)	.711
	Low	0.10 (−0.95, 1.15)	.852	0.27 (−0.74, 1.28)	.599
	None	0.11 (−1.05, 1.28)	.850	−0.14 (−1.09, 0.81)	.771

(b) Endpoint models of parent‐, teacher‐, and child‐reported problems, child age 122 months					
Support (reference)	Contrast	Externalizing problems		Internalizing problems	
		Coefficient (95% CI)	*p*	Coefficient (95% CI)	*p*
N‐res. father (high)	Moderate	0.25 (−0.06, 0.57)	.117	0.48 (0.10, 0.86)	.014
	Low	0.28 (−0.16, 0.72)	.209	0.50 (0.15, 0.86)	.006
	None	0.51 (0.17, 0.85)	.003	0.62 (0.27, 0.97)	<.001

*Notes*. Coefficients are standardized with respect to the outcome variables. Intercepts in Trajectory models set at 84 months (midpoint). Models control for child sex, baseline covariates (mother's age at birth of child, education and mental health, family size, coresident grandparent, household income, area deprivation), and partner separation history. Model fit: Trajectory model RMSEA = 0.07, SRMR = 0.06, Endpoint model RMSEA = 0.07, SRMR = 0.08.

N‐res. = nonresident.

### Grandparent Support and Nonresident Father Support

Regardless of the level of nonresident father support, approximately one in two families received high levels of grandparent support at 34 months, either from maternal grandparents only or from maternal and paternal grandparents. Nonetheless, the prevalence of support provided by both sets of grandparents varied strongly according to nonresident father support class, ranging from only 2% of families not in contact with fathers to 16% of those with high father support (for details, see Table S7).

### Assessing Moderation Effects

In examining our second hypothesis regarding protective effects of high levels of grandparent support, we combined Moderate, Low, and No nonresident father support classes to create a single group (Weaker), to contrast with High nonresident father support. This approach was not only justified by our earlier findings of similar estimated effects for the three weaker father support classes, but also helped address problems relating to low statistical power for small subgroups. We used Trajectory and Endpoint models to estimate main effects of nonresident father support (reference category, High) and grandparent support (reference category, Low), together with interaction terms for grandparent × nonresident father support. Main effects of Weaker nonresident father support were as expected: in the Trajectory model (Table 4, Part a), it was associated with higher externalizing and internalizing intercepts, and a steeper linear growth in internalizing problems; in the Endpoint model (Table 4, Part b) it was associated with higher externalizing and internalizing problems at 122 months. No main effect of any grandparent support class was statistically significant.

**Table 4:** Nonresident Father and Grandparent Support: Associations with Children's Externalizing and Internalizing Problems (*n* = 352)

(a) Trajectory model of parent‐reported problems (46–122 months)					
		Externalizing problems		Internalizing problems	
		Intercept		Intercept	
Support (reference)	Contrast	Coefficient (95% CI)	*p*	Coefficient (95% CI)	*p*
N‐res father (High)	Weaker (Moderate/Low/None)	1.12 (0.39, 1.85)	.003	0.89 (0.29, 1.49)	.004
Grandparent (Low)	Moderate maternal	0.14 (−0.49, 0.77)	.667	0.07 (−0.34, 0.49)	.736
	High maternal	0.38 (−0.25, 1.00)	.243	0.27 (−0.28, 0.81)	.338
	Maternal and paternal	0.70 (−0.11, 1.52)	.090	0.27 (−0.29, 0.82)	.350
Interactions	Mod. Mat. Gpar × Weaker N‐res. father	−0.44 (−1.30, 0.41)	.311	−0.35 (−1.04, 0.34)	.322
	High Mat. Gpar × Weaker N‐res. father	−0.68 (−1.54, 0.18)	.122	−0.66 (−1.43, 0.11)	.092
	Mat. & Pat. Gpar × Weaker N‐res. father	−1.45 (−2.60, −0.31)	.013	−1.52 (−2.34, −0.70)	<.001
		Linear slope		Linear slope	
		Coefficient (95% CI)	*p*	Coefficient (95% CI)	*p*
N‐res father (High)	Weaker (Moderate/Low/None)	0.32 (−0.51, 1.15)	.451	0.76 (0.08, 1.43)	.028
Grandparent (Low)	Moderate maternal	−0.23 (−0.93, 0.47)	.520	0.37 (−0.19, 0.92)	.195
	High maternal	−0.07 (−0.82, 0.67)	.845	0.19 (−0.40, 0.79)	.523
	Maternal and paternal	−0.23 (−1.01, 0.55)	.559	0.32 (−0.33, 0.98)	.336
Interactions	Mod. Mat. Gpar × Weaker N‐res. father	−0.20 (−1.20, 0.80)	.692	−0.34 (−1.17, 0.50)	.430
	High Mat. Gpar × Weaker N‐res. father	−0.25 (−1.24, 0.74)	.618	−0.30 (−1.11, 0.51)	.465
	Mat. & Pat. Gpar × Weaker N‐res. father	−0.32 (−1.63, 0.99)	.633	−1.17 (−2.12, −0.21)	.017
		Quadratic slope		Quadratic slope	
		Coefficient (95% CI)	*p*	Coefficient (95% CI)	*p*
N‐res father (High)	Weaker (Moderate/Low/None)	0.56 (−1.60, 2.72)	.611	−0.96 (−3.07, 1.16)	.376
Grandparent (Low)	Moderate maternal	0.54 (−1.53, 2.60)	.611	−0.41 (−2.02, 1.20)	.619
	High maternal	0.77 (−1.09, 2.63)	.416	−0.49 (−2.19, 1.21)	.575
	Maternal and paternal	−0.31 (−2.04, 1.41)	.723	0.08 (−1.73, 1.90)	.930
Interactions	Mod. Mat. Gpar × Weaker N‐res. father	−1.08 (−3.89, 1.72)	.450	1.41 (−1.27, 4.10)	.302
	High Mat. Gpar × Weaker N‐res. father	−0.63 (−3.08, 1.82)	.613	1.05 (−1.51, 3.60)	.421
	Mat. & Pat. Gpar × Weaker N‐res. father	0.41 (−2.62, 3.44)	.790	1.64 (−1.22, 4.49)	.261

(b) Endpoint model of parent‐, teacher‐, and child‐reported problems at 122 months					
Support (reference category)	Contrast	Externalizing problems		Internalizing problems	
		Coefficient (95% CI)	*p*	Coefficient (95% CI)	*p*
N‐res father (High)	Weaker (Moderate/Low/None)	1.09 (0.28, 1.90)	.009	1.30 (0.65, 1.95)	<.001
Grandparent (Low)	Moderate maternal	0.12 (−0.60, 0.84)	.745	0.29 (−0.33, 0.90)	.365
	High maternal	0.22 (−0.45, 0.90)	.520	0.16 (−0.46, 0.78)	.608
	Maternal and paternal	0.29 (−0.47, 1.04)	.454	0.64 (−0.03, 1.31)	.060
Interactions	Mod. Mat. Gpar × Weaker N‐res. father	−0.79 (−1.75, 0.18)	.108	−0.81 (−1.69, 0.07)	.070
	High Mat. Gpar × Weaker N‐res. father	−0.76 (−1.67, 0.16)	.105	−0.66 (−1.41, 0.10)	.087
	Mat. & Pat. Gpar × Weaker N‐res. father	−1.14 (−2.24, −0.04)	.042	−1.79 (−2.84, −0.74)	.001

*Notes*. Coefficients are standardized with respect to the outcome variables. All models control for child sex, baseline covariates (mother's age at birth of child, education and mental health, family size, coresident grandparent, household income, area deprivation), and partner separation history. Model fit: Trajectory model RMSEA = 0.07, SRMR = 0.06; Endpoint model RMSEA = 0.07, SRMR = 0.07.

Gpar = grandparent; Mat. = maternal; N‐res. = nonresident; Pat = paternal.

Interaction term effects on Trajectory intercepts and linear slopes, and on Endpoint outcomes were negative, indicating that higher levels of grandparent support were protective against Weaker nonresident father support. Interaction terms for Maternal and Paternal grandparent support were statistically significant (*p* < .05), for both Trajectory model intercepts and the internalizing problem linear slope, and for both Endpoint model outcomes. Interactions for High Maternal grandparent support on the Trajectory model internalizing intercept and Endpoint internalizing outcome bordered statistical significance (*p* < .1).

To explore our third hypothesis concerning the greater protective effect of support from both sets of grandparents as compared to support from maternal grandparents only, we ran models again after resetting the grandparent support reference category from Low to the more normative class, High Maternal (not shown in table). Interaction terms for Maternal and Paternal grandparent support × Weaker nonresident father support in relation to internalizing problem levels were statistically significant (effects on Trajectory internalizing intercept: −0.86, *p* = .020 and linear slope −0.87, *p* = .037; on Endpoint internalizing outcome −1.13, *p* = .016). Effects on externalizing problems were not statistically significant, however. Thus, Maternal and Paternal grandparent support afforded children experiencing Weaker nonresident father support more protection against developing internalizing problems, compared to high support from Maternal grandparents alone.

## Discussion

Our study of families headed by a single mother found that children had fewer school‐age externalizing and internalizing problems when they had a nonresident father who was highly supportive of both mother and child in early childhood. The study also found that high levels of grandparent support in early childhood buffered the adverse effects of weaker nonresident father support, with most protection offered by support involving both maternal and paternal grandparents. Findings drew on data from a representative population sample; and were robust to adjustment for a range of confounders including mothers' mental health and family socioeconomic status. They strengthen previous research on single mother families using cross‐sectional data or a limited follow‐up period (Adamsons & Johnson, 2013; Choi et al., 2014; Jackson et al., 2015), by indicating longer‐term effects of early nonresident father support extending into middle childhood and the protective role of grandparents in fostering family resilience. Our findings increase understanding of the role of extended family support for single parents in countries outside the United States with a stronger welfare safety net (Hakovirta & Jokela, 2019).

### Nonresident Father Support

Our novel typology of nonresident father support revealed a close positive association between the quality of the couple relationship and the more often‐studied fathers' investment in their young child (Adamsons & Johnson, 2013). Unlike two studies of families with older children (Elam et al., 2016; Modecki et al., 2015), we did not identify a group with high father involvement and interparental conflict, perhaps because relationship problems deter fathers' involvement more readily after a relatively short period of investment in a child's development. Nonetheless, we found that different patterns of weaker father support, as well as no contact, all had similar negative effects on children's adjustment when compared to families experiencing the highest level of support. Although statistical power may have limited our ability to detect differences, our findings hint at the importance of maintaining a certain threshold level of couple interaction quality and father involvement in early childhood. Our findings also suggest a trade‐off between the benefits of fathering for children's socialization and the undermining effects of couple relationship and coparenting tensions, where the combined effect may resemble that produced by no father contact. While joint custody and shared parenting have grown in popularity in many countries, a recent review highlights the risks posed by interparental conflict to children in these arrangements (Baude et al., 2019).

We found larger effect sizes than is typical for other studies considering nonresident father's child‐directed support, interparental conflict, or coparenting separately (Adamsons & Johnson, 2013; Teubert & Pinquart, 2010). This suggests a cumulative effect of nonresident fathers' support for mother and child, reinforcing the theoretical justification for studying them in combination and suggesting a wider array of possible pathways to child adjustment than research focusing solely on fathers' child‐directed support. Mothers' parenting may be compromised by relationship tensions at a critical stage in early childhood, adding to the effect of couple tensions on fathers' involvement (Stover et al., 2016). Additional potential mechanisms not involving parenting include direct effects of relationship tensions and unsupportive coparenting on children's behavior problems, emotional insecurity, and stress regulation (Harold & Sellers, 2018; Parkes et al., 2019). In the longer term, couple tensions may also hasten parental re‐partnering, with further repercussions for child adjustment (Berger et al., 2018).

### Grandparent Support

The buffering effects we identified for grandparent support echo a number of cross‐sectional studies of all family types, which found that grandparent support reduced the effects of poverty, maternal depression, and poor parenting on children's adjustment (Akhtar et al., 2017; Barnett et al., 2010; Silverstein & Ruiz, 2006). Plausible mechanisms include effects on both mothers and children. Grandparents' financial and in‐kind assistance, coupled with their own experience with raising children, may provide single mothers with critical practical and emotional resources (Barnett et al., 2010). Close emotional bonds developed with grandchildren may allow grandparents to act as dependable surrogate attachment figures (Harper & Ruicheva, 2010; Silverstein & Ruiz, 2006). Our measure of support captured instrumental, rather than emotional support, with further research needed to tease out the effects of different aspects of grandparent care.

Findings suggest the greater value to families of receiving support from both sets of grandparents, rather than (as was typical) maternal grandparents alone. It is not clear whether this is because support levels were higher for families helped by both sets of grandparents, or whether paternal grandparent support has a special role. We were unable to assess the overall level of support from all grandparents due to the response scales used, but findings are consistent with the idea that help from paternal as well as maternal grandparents provides the most robust safety net. Paternal grandparents might also facilitate the child's attachment to a nonresident father (Doyle et al., 2010; Ryan et al., 2008).

### Strengths and Limitations

Our study has some limitations, notably reliance on data collected from mothers regarding father and grandparent support: the perspectives of fathers and children would be valuable additions to future research. Reports of support from nonresident father are typically higher than mothers, and most divergent where there is interparental conflict (Coley & Morris, 2002). Measures of financial provision captured regularity rather than amount; and father involvement was based mainly on contact frequency although this has been shown to be strongly associated with emotional closeness (Nixon et al., 2012). Similarly, our measures of grandparent contact are likely to indicate, albeit imperfectly, the quality of relationships with the mother and child (Mueller & Elder, 2003). Most parents in our sample had already separated by the time the child was 10 months old, preventing exploration of departure timing as a possible moderator. Equally, we have not been able to compare temporary with permanent separations. Future work should explore additional potential moderators of nonresident father support, including grandparent coresidence and family socioeconomic status, explored by few existing studies (Adamsons & Johnson, 2013). Generalizability of our findings may be limited by sample attrition: although the use of survey weights considerably reduced this risk. Generalizability may also be limited by the largely white sample. In choosing to focus on single mothers, we have selected the majority group of single parents: there were too few single fathers to analyze separately, but future studies should try to incorporate this overlooked group. A causal interpretation is bolstered by our two modeling approaches, which had broadly consistent findings. Trajectory models found weaker forms of nonresident father support predicted internalizing problem growth, providing stronger evidence for a causal relationship than effects of support on overall problem levels (intercepts), which could reflect shared variance (McLanahan et al., 2013). Models incorporating outcome information from teachers and children reduce the threat of shared variance to a causal interpretation. Nonetheless, our models assume that nonresident father support will affect child adjustment. Few previous studies have tested bidirectionality: however, two find clearer evidence for effects of fathers on children rather than the reverse (Flouri et al., 2015; Levine Coley & Medeiros, 2007). Our estimates of causal effects also assume no unmeasured confounding, but it is possible that additional factors, such as a mother's personality, affect both support and child outcomes.

## Conclusions

Our typology of nonresident father support suggests that interventions directed at improving children's adjustment by targeting multiple inter‐related aspects of fathering, including coparenting, are likely to be more fruitful than efforts more narrowly focused on fathers' visitation arrangements and/or financial provision. Maintaining a good relationship between mother and father is likely to be key, although maternal gatekeeping of father involvement may relate to justifiable concerns over fathers' parenting competence (Arditti et al., 2019). Several interventions for families transitioning to divorce or postdivorce with a core couple component focusing on relationship skills, or a couple component to supplement parenting skills training, have yielded promising results in relation to child adjustment (Harold & Sellers, 2018). However, many children in our study lacked any contact with the father from an early age. Here, relationships education to help avoid acrimonious splits, and parenting education to encourage joint responsibility for bringing up children and coparenting, might be valuable measures. Although grandparents may provide a vital protective function, they may also need support to maintain access and renegotiate their role after their adult child has separated from his/her partner (O'Dwyer et al., 2012). Yet despite the potential for grandparents to offer close emotional support in addition to “wrap‐around” childcare, grandparent care is unlikely to be a sufficient or universal solution. Alternative sources of support for single mothers including peer support groups, professional advice on parenting from health professionals and high‐quality center‐based childcare are also likely to benefit children's adjustment (Gomajee et al., 2018; Taylor & Conger, 2017).

## Note

This research was supported by Medical Research Council grants MC_UU_12017/11, MC_UU_12017/12, MC_UU_12017/14, and MC_PC_13027, and by Chief Scientist Office Grants SPHSU11, SPHSU12, and SPHSU14. The Growing Up in Scotland study is funded by the Scottish Government. The funder had no part in: the design and conduct of the study; collection, management, analysis, and interpretation of the data; or preparation, review, and approval of the manuscript. We thank Paul Bradshaw and the team at ScotCen Social Research for data collection, and we are grateful to the families who participated in the study.

## Supporting information


**Appendix S1**: Supporting information
