# Supplementary material for: Nonresident Fathers' and Grandparents' Early Years Support and Middle Childhood Socio‐Emotional Adjustment
Source: J Marriage Fam. 2021 Jan 25;83(2):358–74. doi: 10.1111/jomf.12752 (PMC8629113; doi:10.1111/jomf.12752)
Supplement: Supplementary file 1 — Appendix S1: Supporting information [file JOMF-83-358-s001.docx]

**Supplementary File ONLINE ONLY**

**Table S1 Characteristics of the Support Sample and the Analysis Sample**

|  |  | **Support sample** | **Analysis sampl**e |
| --- | --- | --- | --- |
|  | *n* | *648* | *352* |
| **Characteristics at 10-month baseline** | | % or mean (SE) | % or mean (SE) |
| Child sex | Male | 52.9 | 54.1 |
| Mother's age | under 20 | 24.9 | 24.4 |
|  | 20 to 29 | 52.4 | 50.9 |
|  | 30 plus | 22.8 | 24.7 |
| Mother's education | no qualifications | 17.5 | 17.8 |
|  | lower level Standard Grades | 10.8 | 8.6 |
|  | upper level Standard Grades | 38.5 | 38.8 |
|  | Highers | 25.4 | 28.4 |
|  | Degree | 7.9 | 6.4 |
| Mother's mental health |  | 48.1 (0.5) | 48.0 (0.7) |
| Household income quintile | 1 | 58.9 | 57.7 |
|  | 2 | 22.8 | 23.8 |
|  | 3 | 10.7 | 12.3 |
|  | 4 | 5.5 | 4.0 |
|  | 5 | 2.2 | 2.3 |
| Area deprivation quintile | q1 (least deprived) | 4.5 | 4.0 |
|  | q2 | 10.8 | 12.4 |
|  | q3 | 16.4 | 16.8 |
|  | q4 | 23.1 | 20.0 |
|  | q5 (most deprived) | 45.3 | 46.9 |
| Family size | 2 or more children | 45.0 | 47.1 |
| Grandparent in household | yes | 19.3 | 19.6 |
| **Partner separation history** | |  |  |
| Father left household | between child 22 and 34 months | 9.8 | 11.7 |
|  | between 10 and 22 months | 10.9 | 11.5 |
|  | between birth and 10 months | 10.2 | 10.1 |
|  | separated before birth/never together | 69.1 | 66.7 |

Note: Support sample figures use age 34 month survey weights, Analysis sample figures use age 122 month survey weights

**Table S2 Non-resident father support at child age 34 months: details of items**

| **Aspect of support** | **Question addressed to child’s mother** | **Response** | **Mean (SE)** |
| --- | --- | --- | --- |
| **Involvement** | How much interest would you say (Child's name)'s natural father shows in (him/her)? | (1) very interested, (2) somewhat interested, (3) not very interested, (4) not at all interested | 1.50 (0.04) |
|  | How often does (Child's name) usually see his/her natural father at the moment? | (1) every day, (2) 5-6 times a week, (3) 3-4 times a week, (4) once or twice a week, (5) less often than once a month, (7) never | 3.20 (0.09) |
|  | How often does (Child's name) have contact with his/her natural father by telephone, text message, email or letters? | (1) every day, (2) 5-6 times a week, (3) 3-4 times a week, (4) once or twice a week, (5) less often than once a month, (7) never | 3.89 (0.14) |
|  | How often, if at all, does (Child's name)'s natural father have him/her to stay overnight? | (1) every day, (2) 5-6 times a week, (3) 3-4 times a week, (4) once or twice a week, (5) less often than once a month, (7) never | 5.48 (0.08) |
|  | How often, if at all, does (Child's name)'s natural father take (Child's name) on outings or daytrips? | (1) every day, (2) 5-6 times a week, (3) 3-4 times a week, (4) once or twice a week, (5) less often than once a month, (7) never | 4.88 (0.07) |
| **Financial provision** | How often, if at all, does (Child's name)'s natural father buy toys, clothes or equipment for (Child's name) apart from on special  occasions like birthdays? | (1) every day, (2) 5-6 times a week, (3) 3-4 times a week, (4) once or twice a week, (5) less often than once a month, (7) never | 5.39 (0.07) |
|  | Does he currently contribute any money to (Child's name)’s maintenance through (a) Child Support Agency or another formal or legal agreement, (b) any other method? (a and b separate items combined here) | (1) Yes, regular payments, (2) Yes, irregular payments, (3) No | 1.24 (0.05) |
| **Relationship with mother** | How would you describe your relationship with (Child's name)'s father? | (1) very good. (2) fairly good, (3) neither good nor bad, (4) fairly bad, (5) very bad | 2.46 (0.07) |
|  | When you have a serious disagreement with (Child's name)’s natural father, how often do you discuss your disagreements calmly? | (1) often, (2) sometimes, (3) hardly ever, (4) never | 2.08 (0.06) |
|  | When you have a serious disagreement with (Child's name)’s natural father, how often do you reach a compromise? | (1) often, (2) sometimes, (3) hardly ever, (4) never | 1.88 (0.05) |
| **Coparenting** | How often do you talk about (Child's name) with his/her natural father? | (1) several times a week, (2) about once a week, (3) a few times a month, (4) several times a year, (5) once or twice in child's life, (6) not at all | 1.85 (0.07) |
|  | When you make major decisions about (Child's name), how often do you ask (Child's name)’s natural father for his views? | (1) never/almost never, (2) rarely, (3) sometimes, (4) often (5) always/almost always | 3.30 (0.08) |

Note: SE = linearized standard error.

**Latent Class Analysis (LCA) of indicators of non-resident father (NRF) support**

Latent class analysis was performed using Mplus version 8, allowing for the complex survey design, with missing outcome data handled using Full Information Maximum Likelihood estimation. LCA modelled 12 indicators of NRF support as ordered (3-point) categorical variables (see Methods). Various model fit statistics were used to help identify the optimum number of classes, together with considerations of the smallest class size and posterior probabilities of class membership. Smaller Akaike Information Criteria (AIC) and Bayesian Information Criteria (BIC) values are preferable, while Entropy values should be close to 1. The Lo, Mendell and Rubin Likelihood Ratio Test (LMR) test indicated whether a model had a better fit than the model with one fewer class. Table S3 shows that a three-class model provided the optimum solution.

**Table S3 LCA of non-resident father support at 34 months: Model fit statistics**

| Number of classes | Log likelihood | AIC | BIC | Entropy | LMR p value | Smallest class |
| --- | --- | --- | --- | --- | --- | --- |
| 1 | -5162.310 | 10372.619 | 10471.925 |  |  |  |
| 2 | -4494.483 | 9086.966 | 9289.714 | 0.909 | 0.0000 | 38.0 |
| **3** | **-4385.392** | **8918.783** | **9224.975** | **0.838** | **0.0126** | **27.2** |
| 4 | -4318.223 | 8834.446 | 9244.081 | 0.850 | 0.3057 | 13.4 |

## Note: AIC = Akaike information criterion, BIC = Bayesian information criterion, LMR = Lo–Mendell–Rubin Adjusted Likelihood Ratio Test. Bold figures indicate the selected three-class solution.

**Table S4 Grandparent support at child age 34 months: details of items**

|  |  | **Maternal grandparents** | **Paternal grandparents** |
| --- | --- | --- | --- |
| **Type of support** | **Responses to all items** | Mean (SE) | Mean (SE) |
| Look after (Child's name) for an hour or more during the day | (1) every day or almost every day, (2) at least once a week, (3) at least once a month, (4) at least once every three months, (5) less than once every three months, (6) never | 2.97 (0.08) | 5.79 (0.04) |
| Babysit for (Child's name) during the evening |  | 3.95 (0.07) | 5.83 (0.03) |
| Have (Child's name) to stay overnight |  | 3.93 (0.08) | 5.82 (0.03) |
| Take (Child's name) on outings or daytrips without you (or, where applicable) your partner |  | 3.97 (0.07) | 5.81 (0.03) |
| Buy toys, clothes or equipment for (Child's name) apart from on special occasions like birthdays |  | 3.34 (0.06) | 5.73 (0.04) |

Note: SE = linearized standard error

**Latent class analysis of grandparent support**

A latent class analysis was performed on five indicators of grandparent support from maternal, and paternal grandparents, as outlined above for the LCA of non-resident father support indicators. Each indicator of grandparent support was modelled as an ordered (three-point categorical variable), see Methods. Table S5 shows that a four-class model provided the optimum solution.

**Table S5 Latent class analysis of grandparent support at 34 months: Model fit statistics**

| Number of classes | Log likelihood | AIC | BIC | Entropy | LMR p value | Smallest class (%) |
| --- | --- | --- | --- | --- | --- | --- |
| 1 | -4156.66 | 8373.319 | 8507.536 |  |  | 100 |
| 2 | -3640.097 | 7346.194 | 7493.833 | 0.838 | <.001 | 41.8 |
| 3 | -3255.429 | 6582.858 | 6743.918 | 0.923 | <.001 | 9.4 |
| **4** | **-3147.672** | **6373.343** | **6547.825** | **0.864** | **<.001** | **9.5** |
| 5 | -3103.235 | 6290.47 | 6478.374 | 0.881 | 0.473 | 3.2 |

## Note: AIC = Akaike information criterion, BIC = Bayesian information criterion, LMR = Lo–Mendell–Rubin Adjusted Likelihood Ratio Test. Bold figures indicate the selected four-class solution.

**Table S6 Children’s Externalising and Internalising problems from 46 to 122 months in the analysis sample, and according to Non-resident Father Support class**

| 1. **Externalising problems** | | |  |  |  |  |  |  |
| --- | --- | --- | --- | --- | --- | --- | --- | --- |
|  |  |  |  | **Non-resident father support (child 34 months)** | | | |  |
| Child age | Source of information |  | All  (n=352) | None (n=99) | Low  (n=67) | Moderate (n=80) | High (n=106) | Difference *p*-value |
| 46 months | parent | Mean (SE) | 6.45 (0.20) | 7.41 (0.39) | 6.52 (0.45) | 6.48 (0.37) | 5.44 (0.32) | <.001 |
|  |  | % abnormal/borderline | 27 | 38 | 29 | 28 | 15 | 0.016 |
| 58 months | parent | Mean (SE) | 6.51 (0.19) | 7.68 (0.37) | 6.30 (0.50) | 6.73 (0.36) | 5.35 (0.28) | <.001 |
|  |  | % abnormal/borderline | 28 | 39 | 28 | 27 | 18 | 0.019 |
| 70 months | parent | Mean (SE) | 6.25 (0.19) | 7.28 (0.31) | 6.47 (0.54) | 6.34 (0.41) | 4.98 (0.36) | <.001 |
|  |  | % abnormal/borderline | 25 | 33 | 32 | 22 | 15 | 0.028 |
| 94 months | parent | Mean (SE) | 6.34 (0.20) | 7.44 (0.39) | 6.12 (0.49) | 6.44 (0.40) | 5.34 (0.34) | <.001 |
|  |  | % abnormal/borderline | 26 | 36 | 29 | 30 | 11 | 0.002 |
| 122 months | parent | Mean (SE) | 5.39 (0.31) | 6.97 (0.48) | 5.82 (0.51) | 5.80 (0.39) | 4.25 (0.41) | <.001 |
|  |  | % abnormal/borderline | 23 | 37 | 17 | 26 | 10 | 0.002 |
| 122 months | teacher | Mean (SE) | 4.49 (0.41) | 4.85 (0.67) | 3.79 (0.98) | 5.90 (1.08) | 3.39 (0.59) | 0.316 |
|  |  | % abnormal/borderline | 20 | 23 | 18 | 34 | 9 | 0.118 |
| 122 months | child | Mean (SE) | 1.49 (0.05) | 1.57 (0.09) | 1.43 (0.10) | 1.53 (0.08) | 1.42 (0.06) | 0.235 |
|  |  | % abnormal/borderline | N/A | N/A | N/A | N/A | N/A |  |

| 1. **Internalising problems** | | |  |  |  |  |  |  |
| --- | --- | --- | --- | --- | --- | --- | --- | --- |
|  |  |  |  | **Non-resident father support (child 34 months)** | | | |  |
| Child age | Source of information |  | All  (n=352) | None (n=99) | Low  (n=67) | Moderate (n=80) | High (n=106) | Difference *p*-value |
| 46 months | parent | Mean (SE) | 2.84 (0.16) | 3.20 (0.28) | 2.81 (0.34) | 2.75 (0.35) | 2.56 (0.26) | 0.121 |
|  |  | % abnormal/borderline | 10 | 10 | 9 | 13 | 7 | 0.670 |
| 58 months | parent | Mean (SE) | 2.72 (0.19) | 3.34 (0.37) | 2.83 (0.30) | 2.52 (0.42) | 2.23 (0.25) | 0.005 |
|  |  | % abnormal/borderline | 9 | 9 | 10 | 12 | 5 | 0.447 |
| 70 months | parent | Mean (SE) | 3.01 (0.19) | 3.45 (0.34) | 2.95 (0.39) | 2.94 (0.37) | 2.64 (0.31) | 0.063 |
|  |  | % abnormal/borderline | 11 | 10 | 11 | 16 | 7 | 0.353 |
| 94 months | parent | Mean (SE) | 3.65 (0.27) | 4.46 (0.48) | 3.70 (0.47) | 3.95 (0.60) | 2.61 (0.39) | 0.005 |
|  |  | % abnormal/borderline | 19 | 29 | 15 | 23 | 8 | 0.014 |
| 122 months | parent | Mean (SE) | 4.18 (0.33) | 4.58 (0.45) | 4.47 (0.47) | 4.72 (0.59) | 2.73 (0.33) | 0.004 |
|  |  | % abnormal/borderline | 22 | 26 | 23 | 29 | 11 | 0.058 |
| 122 months | teacher | Mean (SE) | 2.72 (0.28) | 3.50 (0.56) | 2.16 (0.61) | 3.20 (0.64) | 1.79 (0.29) | 0.026 |
|  |  | % abnormal/borderline | 7 | 15 | 6 | 6 | 0 | 0.038 |
| 122 months | child | Mean (SE) | 0.13 (0.07) | 0.26 (0.15) | 0.23 (0.13) | 0.14 (0.15) | -0.08 (0.09) | 0.043 |
|  |  | % abnormal/borderline | N/A | N/A | N/A | N/A | N/A |  |

Note: Abnormal/borderline cut-offs use recommended values for the Strengths and Difficulties questionnaire (SDQ). N/A Not applicable, because child items did not use the SDQ**.** Figures allow for clustered sampling and survey weights.

**Table S7 Association between non-resident father support and grandparent support**

|  |  | **Non-resident father support class** | | | |  |
| --- | --- | --- | --- | --- | --- | --- |
| **Grandparent support class** |  | No support | Low support | Moderate support | High support | Total |
| High maternal and paternal | column % | 1.6 | 9.2 | 9.3 | 16.3 | 9.2 |
|  | *n* | *2* | *7* | *8* | *17* | *34* |
|  |  |  |  |  |  |  |
| High maternal | column % | 56.3 | 44.1 | 33.1 | 37.5 | 42.8 |
|  | *n* | *52* | *28* | *25* | *34* | *139* |
|  |  |  |  |  |  |  |
| Moderate maternal | column % | 29.2 | 31.9 | 52.3 | 29.2 | 35.5 |
|  | *n* | *32* | *24* | *41* | *34* | *131* |
|  |  |  |  |  |  |  |
| No support | column % | 13.0 | 14.8 | 5.3 | 17.1 | 12.6 |
|  | *n* | *13* | *8* | *6* | *21* | *48* |
|  |  |  |  |  |  |  |
| Total | column % | 100.0 | 100.0 | 100.0 | 100.0 | 100.0 |
|  | *n* | *99* | *67* | *80* | *106* | *352* |

Note: “Weaker” non-resident father support (as used in moderation analyses) =No support+Low support+Moderate support (n=246)
